# Development and evaluation of a multiplex serodiagnostic bead assay (BurkPx) for accurate melioidosis diagnosis

**DOI:** 10.1371/journal.pntd.0011072

**Published:** 2023-02-08

**Authors:** Erik W. Settles, Derek Sonderegger, Austin B. Shannon, Kimberly R. Celona, Rachel Lederer, Jinhee Yi, Courtney Seavey, Kyle Headley, Mimi Mbegbu, Maxx Harvey, Mitch Keener, Chris Allender, Heidie Hornstra, Fernando P. Monroy, Celeste Woerle, Vanessa Theobald, Mark Mayo, Bart J. Currie, Paul Keim

**Affiliations:** 1 The Pathogen and Microbiome Institute, Northern Arizona University, Flagstaff, Arizona, United States of America; 2 Department of Biological Sciences, Northern Arizona University, Flagstaff, Arizona, United States of America; 3 Department of Mathematics and Statistics, Northern Arizona University, Flagstaff, Arizona, United States of America; 4 Global and Tropical Health Division, Menzies School of Health Research, Charles Darwin University, Darwin, Northern Territory, Australia; 5 Infectious Diseases Department and Northern Territory Medical Program, Royal Darwin Hospital, Darwin, Northern Territory, Australia; National University of Singapore, SINGAPORE

## Abstract

*Burkholderia pseudomallei*, the causative agent of melioidosis, is a gram-negative soil bacterium well recognized in Southeast Asia and northern Australia. However, wider and expanding global distribution of *B*. *pseudomallei* has been elucidated. Early diagnosis is critical for commencing the specific therapy required to optimize outcome. Serological testing using the indirect hemagglutination (IHA) antibody assay has long been used to augment diagnosis of melioidosis and to monitor progress. However, cross reactivity and prior exposure may complicate the diagnosis of current clinical disease (melioidosis). The goal of our study was to develop and initially evaluate a serology assay (BurkPx) that capitalized upon host response to multiple antigens. Antigens were selected from previous studies for expression/purification and conjugation to microspheres for multiantigen analysis. Selected serum samples from non-melioidosis controls and serial samples from culture-confirmed melioidosis patients were used to characterize the diagnostic power of individual and combined antigens at two times post admission. Multiple variable models were developed to evaluate multivariate antigen reactivity, identify important antigens, and determine sensitivity and specificity for the diagnosis of melioidosis. The final multiplex assay had a diagnostic sensitivity of 90% and specificity of 93%, which was superior to any single antigen in side-by-side comparisons. The sensitivity of the assay started at >85% for the initial serum sample after admission and increased to 94% 21 days later. Weighting antigen contribution to each model indicated that certain antigen contributed to diagnosis more than others, which suggests that the number of antigens in the assay can be decreased. In summation, the BurkPx assay can facilitate the diagnosis of melioidosis and potentially improve on currently available serology assays. Further evaluation is now required in both melioidosis-endemic and non-endemic settings.

## Author summary

Melioidosis has a wide diversity of symptoms, making laboratory diagnosis essential. The diagnosis of melioidosis is primarily by culture of the causative bacterium, *Burkholderia pseudomallei*, which is problematic for locations with limited laboratory resources and for laboratories inexperienced in handling *B*. *pseudomallei*. Early diagnosis of melioidosis facilitates the specific antimicrobial therapy that optimizes outcomes and substantially lowers mortality. We have developed and evaluated a multiple antigen-antibody serology assay using serial samples from culture confirmed melioidosis patients including the first sample after patient admission, together with non-melioidosis serum controls. Multiantigen reactivity was compared to single antigens and to the indirect hemagglutination (IHA) antibody assay data collected from routine patient testing. The results from testing patients with culture-confirmed melioidosis suggest that using multiple individual antigen assays coupled with models can improve the sensitivity and specificity of serological diagnosis using the same antigens evaluated individually. In addition, all evaluated antigens may not be needed to improve assay performance. Further antigen down selection and evaluation of the utility of this assay is now required in both melioidosis-endemic and non-endemic settings.

## Introduction

Melioidosis is a disease caused by an infection with the Gram-negative soil bacterium *Burkholderia pseudomallei* [1,2]. It is a significant global public health threat with modelling estimating the burden of disease to be 165,000 cases annually, with 89,000 fatalities [3]. Endemic region mortality can range from ~10% in Australia to >40% in Thailand [2]. The highest incidence of melioidosis disease occurs in the *B*. *pseudomallei* endemic regions, which include sub-tropical and tropical regions of the world including northern Australia and Southeast Asia [4]. Inhalation or ingestion of contaminated soil or water, inoculation through a wound, or injection with *B*. *pseudomallei* are the proposed routes of infection with wound inoculation presumed to be the most common. As such, agricultural workers who spend long hours exposed to soil in endemic regions or individuals who are immunocompromised are at a higher risk for infection and disease [2,5,6]. Following exposure to *B*. *pseudomallei*, the symptomology of disease is protean, ranging from flu like symptoms to rapidly progressive sepsis resulting in death [2]. The incubation period has been reported to range from 1 to 21 days, with a median of 4 days between the infecting exposure event and onset of symptoms [7]. Mortality in melioidosis is substantially decreased by use of specific antimicrobial therapy, which is different from the standard empirical therapy for pneumonia and sepsis [2]. Therefore, facilitating earlier diagnosis of melioidosis is critical to optimizing patient outcomes.

Currently, the “gold standard” for diagnosing melioidosis is to employ culture-based techniques to grow *B*. *pseudomallei* from a clinical specimen such as blood, sputum, pus, or urine [1]. This method can be tedious and prolongs time to diagnosis. Although highly specific (100%), the culture technique is slow, and its sensitivity has been estimated to be as low as 60% [8]. The second most widely used clinical diagnostic tool is a serology assay—the indirect hemagglutination assay (IHA). The IHA uses *B*. *pseudomallei* whole cell lysate coated red blood cells to detect patient antibodies to *B*. *pseudomallei* and determine an antibody titer using red blood cell agglutination. The turnaround time for a result is rapid, but the utility is limited for two reasons. Firstly, serology is often negative on admission. When testing the first available serum sample, one study reported a sensitivity of 56%, but subsequent seroconversion increased sensitivity to around 85% [9]. Secondly, in melioidosis-endemic regions positive IHA can reflect past infection with *B*. *pseudomallei* rather than acute disease (melioidosis), with the background seropositivity of the IHA assay reaching over 50% in northeast Thailand [10,11]. Nevertheless, in non-endemic locations without background exposure to *B*. *pseudomallei* a positive IHA has high specificity for acute disease (melioidosis).

A multitude of new serological diagnostic tools have been created, with the aim to improve on IHA for a timely and effective diagnosis of melioidosis, but none is currently approved and routinely used [12–22]. Multiplex assays that incorporate various *B*. *pseudomallei* immunogenic antigens have expanded the power of serological assays in the detection of antibodies to *B*. *pseudomallei*. The 20 *B*. *pseudomallei* proteins used in a 2D microarray detection assay exceeded the sensitivity and specificity of the IHA (86.7% versus 57%, and 97% versus 96%) [16]. Highly immunogenic antigens, like OPS, CPS and HCP1, have been implemented into rapid enzyme linked immunosorbent assays (ELISA) [13].

We sought out immunogenic *B*. *pseudomallei* proteins and carbohydrates previously published and employ a clinically approved Luminex xMAP technology, which is a bead/microsphere-based multiplex immunoassay diagnostic platform, to detect antibodies to *B*. *pseudomallei* that can support a diagnosis of melioidosis. This technology allows simultaneous detection of multiple antigens, including proteins and carbohydrates, in a single assay that has a similar timeframe to ELISA serology assays. We then evaluated the reactivity of single antigens and compared these responses to a method that interprets multiple antigen responses simultaneously. Finally, we identified a smaller subset of antigens for use in future multiplex serology assays.

## Materials and methods

### Ethics statement

This study was approved by the Human Research Ethics Committee of the Northern Territory Department of Health and the Menzies School of Health Research (HREC 02/38 and HREC 2014–2037). The negative serum samples collected in the United States were deemed exempt by the Northern Arizona University Institutional Review Board (IRB). Written formal consent was obtained prior to enrollment and sample collection.

### Patient population and sample collection

Serial venous whole blood was collected from 56 culture-confirmed patients with melioidosis from the Darwin Prospective Melioidosis Study [7]. The melioidosis patients were subdivided into the following clinical categories: 50 were acute only, 6 were chronic (as defined by disease symptoms being present for 2 months or longer before diagnosis), and no patients were activation of latent infection. Samples collected after the acute period do not classify the patients as chronic or latent since blood draws were collected upon patient follow-up when possible. In total 386 blood samples were collected and serum was separated, aliquoted, and stored at -80°C until use. For controls, a single residual serum sample from blood donors was collected by Creative Testing Solutions from Louisiana (n = 200) and California (n = 200). These control samples are referred to as non-melioidosis sera samples, since they are collected in non-endemic or low endemicity areas. However, there is the unlikely theoretical possibility that one or more could have been collected from an individual exposed to *B*. *pseudomallei* when traveling to endemic regions. All serum samples were handled in a biosafety cabinet using standard biological safety level two (BSL2) practices and reactivity assessed using ELISA and multiplex assay protocols were approved by the Northern Arizona University Institutional Biosafety Committee.

### *B*. *pseudomallei* whole cell lysate preparation

In a biological safety level three (BSL3) facility, *B*. *pseudomallei* 1026b was grown on M9 plus case amino acids and LB agar at 37°C for 35–48 hours. Difco M9 minimal salts were prepared (BD) and was supplemented (0.4% glucose, 0.5% case amino acids, 2mM MgSO_4_, 0.1mM CaCl_2_, and 15g/L agar) to induce type VI secretion system proteins [23]. After incubation, single colonies of *B*. *pseudomallei* were scraped and suspended in phosphate buffered saline (PBS) solution, pH 7.4, to yield a turbidity reading of 1.0–1.2 at OD_600_. The bacteria cells suspended in PBS buffer were washed and centrifuged twice at 16,000 x g for 3 minutes at 4°C to pellet the cells. Resulting cell pellets were resuspended in lysis buffer (50mM KH_2_PO_4_, 400mM NaCl, 100mM KCl, 0.5% Triton X-100, and 10mM imidazole), pH 7.4. Lysing was performed by a freeze and thaw technique using liquid nitrogen and 42°C water bath, respectively, repeated three to six times. Whole cell lysate (WCL) proteins were prepared as previously described [24].

### *B*. *pseudomallei* whole cell lysate ELISA and sample selection

Enzyme-Linked Immunosorbent Assays (ELISA) were performed with 400 serum samples from healthy human donors (n = 400) or culture confirmed melioidosis patients (n = 36) on plates coated with *B*. *pseudomallei* 1026b. The ELISA method for the WCL ELISA were performed as previously described [24] with modifications. Briefly, 96 well ELISA plates (Fisher Microfluor 2) were coated with 100μL of WCL at 1.5μg/mL diluted in 1x PBS (Fisher Scientific) and incubated 14–16 hours at 4°C. Following incubation, plates were washed with PBS containing 0.05% TWEEN 20 and then blocked with 1% BSA (Fisher Scientific) diluted in PBS and incubated for 2 hours. The plates were washed once more before application of serum. Each serum sample was diluted in 1% BSA at factors of 1:1,000 up to 1:80,000 and 100μL of the diluted serum was incubated in the plates for 1 hour. The plates were washed again and then incubated with an anti-human IgG or IgM antibody conjugated to horseradish peroxidase (HRP) (Promega) for one hour. A final wash was followed by development with Amplex UltraRed reagent (Life Technologies) and halted with Amplex Stop reagent (Life Technologies) after a determined optimal development time. The plates were read on a Synergy HT Microplate Reader (BioTek). Each serum sample analysed was technically replicated and was compared to a within-assay standard of known concentrations of purified human IgG or IgM, depending on the subtype targeted. Standard curves, averages, and standard deviations of total *B*. *pseudomallei*-reactive IgG and IgM were calculated using five parameter logistic curve fit of the Gen5 software (BioTek).

**Fig 1:**
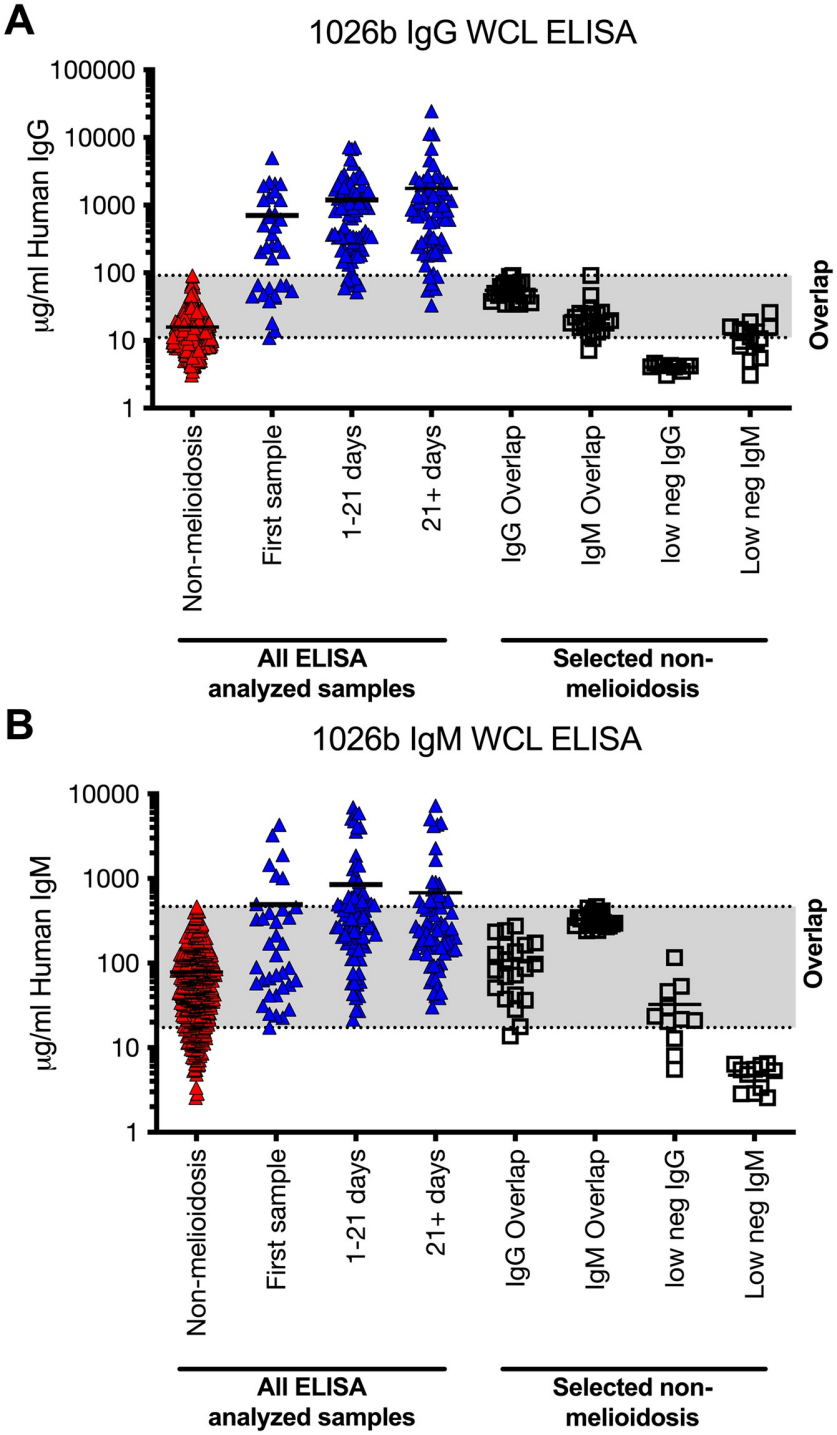
Serum reactivity of melioidosis-confirmed patients and non-melioidosis individuals to *B*. *pseudomallei* whole cell lysate ELISA. Serum IgG (A) or IgM (B) reactivity as determined by *B*. *pseudomallei* 1026b whole cell lysate ELISA. Triangles represent ELISA analyzed serum (n = 585) from 436 individuals (36 melioidosis and 400 non-melioidosis individuals) and squares represent selected serum samples to evaluate potential diagnostic antigens. The overlap between the negative and the positive samples are shown in grey.

Following 1026b WCL ELISA screening of the control and a subset of the melioidosis sample sets, a smaller sample set was selected based upon resulting antibody reactivity (Fig 1). For the non-melioidosis sample set (n = 76), we specifically chose samples that had overlapping antibody reactivity with melioidosis patient samples. These non-melioidosis samples, termed IgG or IgM overlap, included 21 negative overlap sera samples that were cross reactive for IgG and 19 negative overlap samples that were cross reactive for IgM (n = 40). In addition, we selected low *B*. *pseudomallei* 1026b whole cell lysate ELISA cross reactive IgG (n = 18) and IgM (n = 18) serum samples (n = 36). For the melioidosis patient sample set, we included two serum samples from 56 patients (n = 112). The first serum sample (n = 56) was the earliest serum sample collected from the patient when they were admitted at the Royal Darwin hospital. The second serum sample was collected approximately 21 days later (n = 56). The distribution of these samples from the first sample collected from the patient and the days after admission are shown in S1 Fig. Both the selected non-melioidosis and melioidosis serum samples were analyzed by the MAGPIX assay and analysis of these samples are shown in Tables 2–5.

### Antigen purification and conjugation to MAGPLEX microspheres

We purified 46 *B*. *pseudomallei* antigens that included recombinant proteins or purified carbohydrates from *B*. *pseudomallei*. The proteins were initially cloned into an *Escherichia coli* expression plasmid with a histidine tag (his6) on the N-terminus of the protein. Proteins were purified using soluble expression methods and nickel affinity chromatography (HisPrep FF 16/10, GE Life Sciences) using manufacturer’s protocols. However, some of the proteins remained insoluble and we either increased solubility by adding an *E*. *coli* thioredoxin solubility tag paired with the his6 tag or by chemical solubilization using N-Lauroylsarcosine that is compatible with MagPlex microsphere conjugation [25]. The chemical solubilization and purification was performed as previously described [25] and in the protein refolding kit (TB234 12/98, Novagen, Inc, WI, USA) with minor modifications. In brief, inclusion bodies were isolated by centrifugation 10,000 x g for 10 minutes at 4°C. The inclusion bodies were washed four times with Wash Buffer (20 mM Tris-HCl, pH 7.5, 10 mM EDTA, 1% Triton X-100). The washed inclusion body pellet was suspended at 30mg/ml in solubilization buffer (50 mM CAPS, pH 11.0, 0.3% N-Lauroylsarcosine) and incubated for one hour at room temperature. The insoluble protein was removed by centrifugation and the soluble fraction was collected. The solubilized protein was then purified using a nickel affinity column (HisPrep FF 16/10, GE Life Sciences) and eluted using imidazole per the manufacture’s instructions with slight modification. All buffers contained N-Lauroylsarcosine to keep the proteins soluble. Protein concentrations were determined using the Bradford assay with bovine serum albumen (BSA) as a standard (S1 Table). Full length purified protein size was confirmed by SDS-PAGE and western analysis using anti-his6 antibody. Protein purity was determined using SDS-PAGE and SyproRuby stain. The purity was determined by densitometry and purity was determined by ImageJ (S1 Table). Capsular polysaccharide (CPS) and Lipopolysaccharide type A (LPS A) were prepared as previously described [24]. The amount of purified polysaccharide was determined by dry weight after lyophilization. Isolation and lack of cross contamination by other carbohydrates during purification was confirmed using carbohydrate specific monoclonal antibodies, kindly provided by Dr. David AuCoin at the University of Nevada, Reno.

Purified soluble and chemically solubilized proteins were conjugated to different fluorescently labeled MagPlex microspheres (also called bead regions). For proteins conjugation onto the microspheres, the carbodiimide coupling reaction was used that included Sulfo-NHS (N-hydroxysulfosuccinimide) and EDC (1-ethyl-3-(3-dimethylaminopropyl) carbodiimide hydrochloride) (Luminex xMAP Cookbook). We determined the optimal concentration to couple naturally soluble and N-Lauroylsarcosine soluble recombinant proteins to microspheres. Coupling was confirmed using the anti-6x His monoclonal antibody (clone AD1.1.10) biotin conjugate (Abcam) and Streptavidin, R-Phycoerythrin Conjugate (SAPE, Life technologies). For purified carbohydrates conjugation to microspheres, we used the 4-(4,6-dimethoxy[1,3,5] triazin-2-yl)-4-methyl-morpholinium (DMTMM) and desalting columns (GE healthcare) as previously described [26]. Purified carbohydrates LPS A, LPS B, or CPS were coupled to MagPlex microspheres and the coupling was confirmed using LPS- or CPS-specific monoclonal antibodies, kindly provided by Dr. David AuCoin at the University of Nevada, Reno [27–29], goat anti-mouse IgG biotin conjugate (ThermoFisher), and SAPE. All conjugated microspheres were blocked and stored in PBS-TBN buffer (0.01 phosphate, pH 7.4, 138 mM NaCl, 2.7mM KCl, 0.1% BSA, 0.02% Tween-20, and 0.05% sodium azide). A summary of the coupled bead regions are shown in Table 1.

**Table 1:** Purified antigens coupled to bead regions and the associated purpose.

#	Common protein or carbohydrate name	*K96243 locus tag*	*Purpose*	*purification tag(s)*	*Coupling verification method*	*Original reference*
1	GroEL	BPSL2697	Diagnostic	Histidine tag	Anti-his6 mAb	[30]
2	GroS	BPSS0476	Diagnostic	Histidine tag	Anti-his6 mAb	[31]
3	TPX	BPSL2987	Diagnostic	Histidine tag	Anti-his6 mAb	[32]
4	rpIL	BPSL3222	Diagnostic	TRX and Histidine tag	Anti-his6 mAb	[31]
5	Paaz	BPSL3041	Diagnostic	Histidine tag	Anti-his6 mAb	[32]
6	EfTu	BPSL3215	Diagnostic	Histidine tag	Anti-his6 mAb	[32,33]
7	ClpX	BPSL1404	Diagnostic	Histidine tag	Anti-his6 mAb	[32]
8	CPS	none	Diagnostic	Histidine tag	Anti-CPS mAb^1^	[19]
9	1026b WCL	none	Diagnostic	None	Goat serum^2^	[34]
10	Enolase	BPSL2270	Diagnostic	Histidine tag	Anti-his6 mAb	[32]
11	ATP/GTP-binding protein	BPSS1385	Diagnostic	Histidine tag	Anti-his6 mAb	[31]
12	AhpC2	BPSL2748	Diagnostic	Histidine tag	Anti-his6 mAb	[32,33]
13	DNAk	BPSL2827	Diagnostic	Histidine tag	Anti-his6 mAb	[35]
14	AhpC	BPSL2096	Diagnostic	Histidine tag	Anti-his6 mAb	[31]
15	FtsA	BPSS3021	Diagnostic	Histidine tag	Anti-his6 mAb	[24,32]
16	LPS B	none	Diagnostic	Histidine tag	Anti-LPS B mAb^3^	[36]
17	lipoprotein	BPSL0093	Diagnostic	TRX and Histidine tag	Anti-his6 mAb	[31]
18	hypothetical protein	BPSS0135	Diagnostic	Histidine tag	Anti-his6 mAb	n/a
19	NADH	BPSS1769	Diagnostic	Histidine tag	Anti-his6 mAb	[32]
20	IPMS	BPSL1201	Diagnostic	Histidine tag	Anti-his6 mAb	[32]
21	PhAC	BPSL3234	Diagnostic	Histidine tag	Anti-his6 mAb	[32]
22	PDHD	BPSL2299	Diagnostic	Histidine tag	Anti-his6 mAb	[32]
23	MDH	BPSS1722	Diagnostic	Histidine tag	Anti-his6 mAb	[31]
24	LPS A	none	Diagnostic	Histidine tag	Anti-LPSA mAb^4^	[37,38]
25	ABC transporter ATP-binding protein	BPSS1652	Diagnostic	Histidine tag	Anti-his6 mAb	[31]
26	Arg	BPSL1743	Diagnostic	Histidine tag	Anti-his6 mAb	[32]
27	GroEL2	BPSS0477	Diagnostic	Histidine tag	Anti-his6 mAb	[31]
28	hypothetical protein	BPSS0530	Diagnostic	TRX and Histidine tag	Anti-his6 mAb	[31]
29	efta	BPSL2499	Diagnostic	Histidine tag	Anti-his6 mAb	[32]
30	hypothetical protein	BPSL0739	Diagnostic	Histidine tag	Anti-his6 mAb	[31]
31	OmpA	BPSL2522	Diagnostic	TRX and Histidine tag	Anti-his6 mAb	[39]
32	outer membrane porin protein	BPSS2160	Diagnostic	Histidine tag	Anti-his6 mAb	[31]
33	bopE	BPSS1525	Diagnostic	Histidine tag	Anti-his6 mAb	[31]
34	fliC	BPSL3319	Diagnostic	Histidine tag	Anti-his6 mAb	[31]
35	hypothetical protein	BPSS1516	Diagnostic	TRX and Histidine tag	Anti-his6 mAb	[31]
36	ATPase subunit beta	BPSL3396	Diagnostic	Histidine tag	Anti-his6 mAb	[32]
37	FlgK	BPSL0280	Diagnostic	Histidine tag	Anti-his6 mAb	[31]
38	hypothetical protein	BPSL2520	Diagnostic	Histidine tag	Anti-his6 mAb	[31]
39	sucC	BPSL0779	Diagnostic	Histidine tag	Anti-his6 mAb	[24]
40	hypothetical protein	BPSS1850	Diagnostic	Histidine tag	Anti-his6 mAb	[35]
41	hypothetical protein	BPSL2030	Diagnostic	Histidine tag	Anti-his6 mAb	[31]
42	fructan beta-fructosidase	BPSS0542	Diagnostic	Histidine tag	Anti-his6 mAb	[31]
43	lipoprotein	BPSL2705	Diagnostic	Histidine tag	Anti-his6 mAb	[32]
44	ATP synthase F0F1 subunit alpha	BPSl3398	Diagnostic	Histidine tag	Anti-his6 mAb	[32]
45	S-adenosylmethionine synthetase	BPSL0212	Diagnostic	Histidine tag	Anti-his6 mAb	[35]
46	hypothetical protein	BPSL2005	Diagnostic	Histidine tag	Anti-his6 mAb	n/a
47	lipopolysaccharide biosynthesis protein	BPSS0421	Diagnostic	Histidine tag	Anti-his6 mAb	[31]
48	Purified IgG	None	Pos Control	None	Goat anti-human IgG	n/a
49	Purified IgM	None	Pos Control	None	Goat anti-human IgM	n/a

^1^ purified CPS monoclonal antibody was generated previously [27].

^2^ goat serum from a *B*. *pseudomallei* challenged goat with known *B*. *pseudomallei* reactivity was used to confirm conjugation [24].

^3^ purified LPS B monoclonal antibody was generated previously [28].

^4^ purified LPS A monoclonal antibody was generated previously [40].

### MAGPIX multiplex assays

Protein or carbohydrate conjugated microspheres were sonicated and diluted in 1x Blocker BSA solution (Fisher Scientific) and 100μl of microspheres was aliquoted per well (1000 microspheres per region). Microspheres were washed 2X with wash buffer (11.9 mM phosphate, pH 7.4, 137 mM NaCl, 2.7 mM KCl, and 0.05% Tween 20) and the wash buffer was removed after microspheres were bound to a plate magnet. Serum was diluted 1000-fold in 1x Blocker BSA solution and 100 μl diluted serum was added to the microspheres. Serum and microspheres were incubated for two hours with shaking at room temperature. The microspheres were washed three times with wash buffer and 100 μl of 2 μg/ml goat anti-human IgG (Abcam) or 1 μg/ml goat anti-human IgM mu chain (Abcam) biotin conjugate secondary antibody diluted in 1x Blocker BSA were added to the microspheres. The secondary antibody and microspheres were incubated for one hour with shaking at room temperature and washed three times with wash buffer after incubation. Microspheres were then incubated with 4 μg/ml SAPE (Life Technologies) diluted in 1x Blocker BSA. The SAPE and microspheres were incubated for 0.5 hours with shaking at room temperature and washed three times with wash buffer after incubation. Microspheres were suspended in 1x Blocker BSA, and the microspheres were read on a MAGPIX system (Luminex) and the median fluorescent intensity (MFI) units per microsphere region was calculated using xPONENT software (Luminex). The antigens were evaluated in three separate batches, but the antigen reactivity data were merged for comparison.

### Statistical analysis

Statistical analysis was performed using GraphPad Prism version 9 (Graph Pad Software Inc) or the statistical R package version 3.6.0 utilizing the glmnet package version 3.0–3. The specific analysis performed is shown in each figure legend. For the multivariate model generation and data interpretation, two shrinkage based binomial regression models [41] that we trained include 1) the least absolute shrinkage and selection operator (LASSO) model and 2) a Ridge regression (RR) model, both of which are a slight variation to the normal binomial regression analysis. The LASSO method starts with a binomial logistic regression, but an additional penalty term forces the regression coefficients towards zero (and to zero, effectively removing it from the model). This modification allows the ranking of antigens and their specific immune response (IgG or IgM) that contribute to the model. Antigen reactivity data used to generate the models were collected from the MAGPIX multiplex assay and included reactivity from fifty-six melioidosis patients (paired samples—total 112 samples) and 76 selected non-melioidosis samples.

## Results

### Evaluation of sample reactivity to *B*. *pseudomallei* WCL

We selected known negative and positive serum that had different levels of *B*. *pseudomallei* reactivity for antigen screening purposes. To this end, we evaluated negative melioidosis samples collected in the United States and culture-confirmed melioidosis patient serum samples for *B*. *pseudomallei* reactivity using a *B*. *pseudomallei* 1026b whole cell lysate ELISA for IgG and IgM reactivity (Fig 1). For both IgG and IgM reactivity, there was a mean antibody reactivity increase in melioidosis patients relative to the negative samples (IgM 9-fold increase and IgG 83-fold increase) and there was an increase in the antibody reactivity of the melioidosis patient samples collected over time. In addition, the IgG antibody response in melioidosis patients was higher over time compared to the IgM response in the same patient samples (mean reactivity IgM– 714 μg/ml compared IgG 1314 μg/ml). However, there is a clear zone of signal intensity overlap for both IgG and IgM measured responses between negative and melioidosis samples. The overlap of reactive antibody signal could lead to false positive and negative serum samples if using the *B*. *pseudomallei* WCL ELISA to diagnose melioidosis. For IgM, 81% of the first melioidosis samples overlap with the negative sample antibody reactivity and this decreases down to 61% or 71% for later collected samples. For IgG reactivity, a similar but smaller overlap of 37% of collected samples has similar antibody reactivity compared to negative samples for the first sample and the number of sample overlap decreases to 9–10% at later time points. For the negative samples, 83% of the negative samples end up in this overlap reactive zone for IgM and 56% for IgG reactivity. Thus, early melioidosis patient samples collected after admission are more difficult to distinguish from the non-melioidosis serum samples. Using the WCL ELISAs antibody reactivity data and the overlap zone, we selected a set of serum samples (Total 188 samples) from the non-melioidosis group (Fig 1 **squares**, 76 samples) and melioidosis groups (n = 56 individuals, 112 paired samples) to evaluate a multiplex serology assay and perform subsequent analysis. These samples were selected to increase stringency for antigen evaluation and increase the potential to diagnose melioidosis patients quickly after admission.

### Evaluation of antigen conjugated microspheres

**Fig 2:**
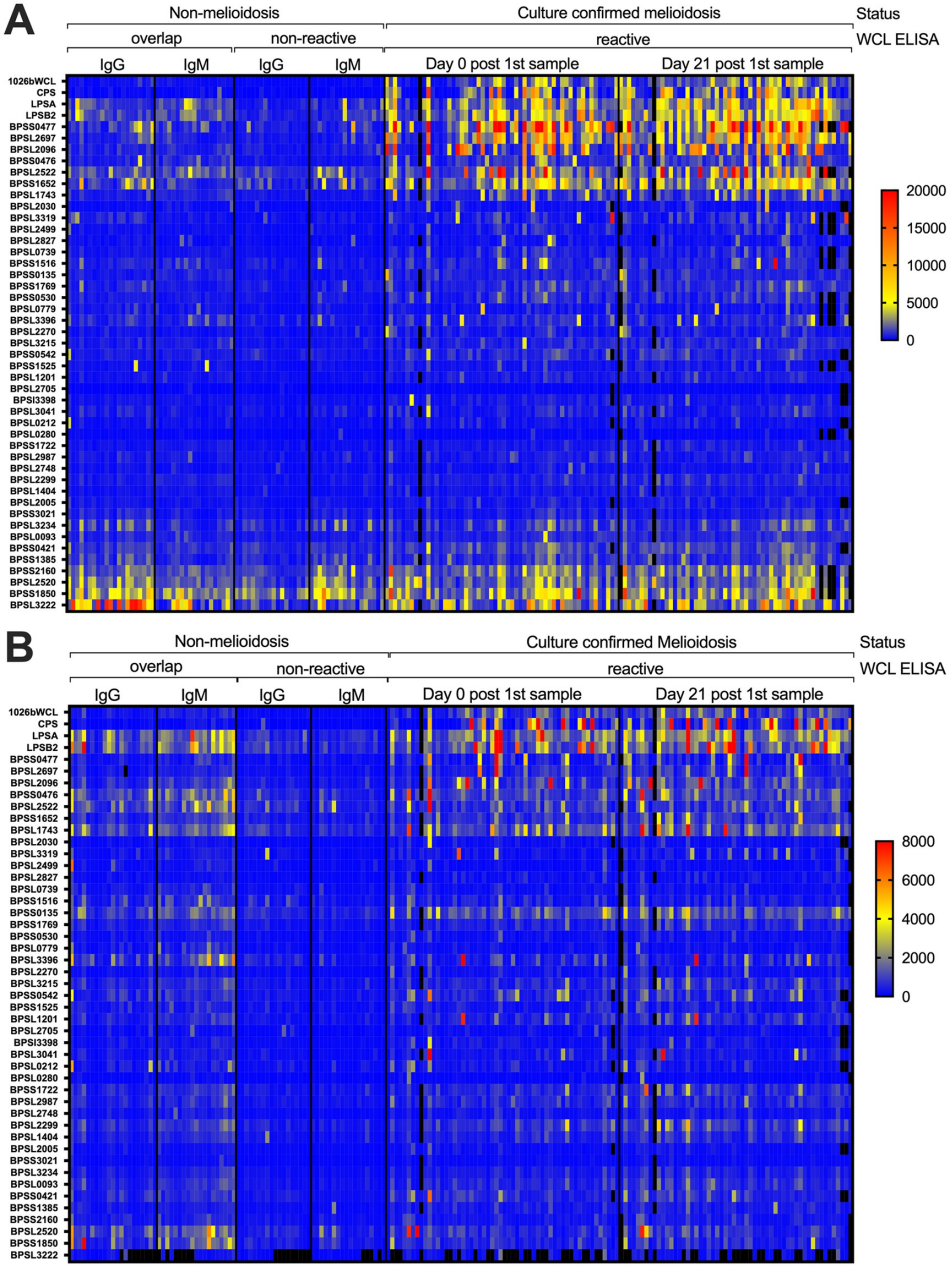
Heat map of MAGPIX multiplexed assay for IgG (A) and IgM (B). Each row represents an individual antigen, and each column represents an individual serum sample (n = 188). The sera are stratified by non-melioidosis negative and melioidosis samples. The negative group is further stratified by the 1026b whole cell lysate (WCL) reactivity (overlap and non-reactive IgG and IgM). The melioidosis serum is grouped by the sample’s collection time after initial sample collection. For the heat map, blue indicates 0 median fluorescent intensity (MFI) for both panels. For IgG panel A, yellow indicates 5,000 MFI and red indicates >20,000 MFI. For IgM panel B, yellow indicates 4,000 MFI and red indicates >8,000 MFI. Black indicates samples where data was not collected.

Several studies have identified diagnostic antigens (Table 1) and in some cases evaluated their sensitivity and specificity. However, we wanted to confirm their antigenicity when attached to MagPlex microspheres and determine their performance in a multivariate assay. To evaluate the antigens, we conjugated purified proteins or carbohydrates to the MagPlex microspheres (Table 1). The selected melioidosis and non-melioidosis serum samples (n = 188) were used to detect patient reactive IgG or IgM antibody responses (Fig 2). For culture confirmed melioidosis samples, reactivity of several antigens for IgM was lower compared to IgG reactivity in the same samples. For IgG and IgM reactivity, a subset of antigens had a lower reactivity in the negative samples and a high reactivity in the melioidosis patient samples at both time points. These antigens include LPSB, CPS, GroEL (BPSL2697), GroEL2 (BPSS0477), AhpC (BPSL2096), OmpA (BPSL2522) and BPSL1652. In contrast, antigens such as BPSL3222, BPSS1850, BPSL2520, and BP2160 had a higher or similar IgG reactivity in non-melioidosis individual sera samples compared to melioidosis-confirmed patient sera samples. These antigens may represent epitopes that are conserved in other pathogens. Furthermore, the antibody response to these antigens was elevated in the reactive negative serum samples, suggesting that these antibody responses could cross react with *B*. *pseudomallei* and contribute to false positive responses. In combination, these cross-reactive antigens may contribute to a higher *B*. *pseudomallei* WCL ELISA overlap for the negative patient samples.

**Fig 3:**
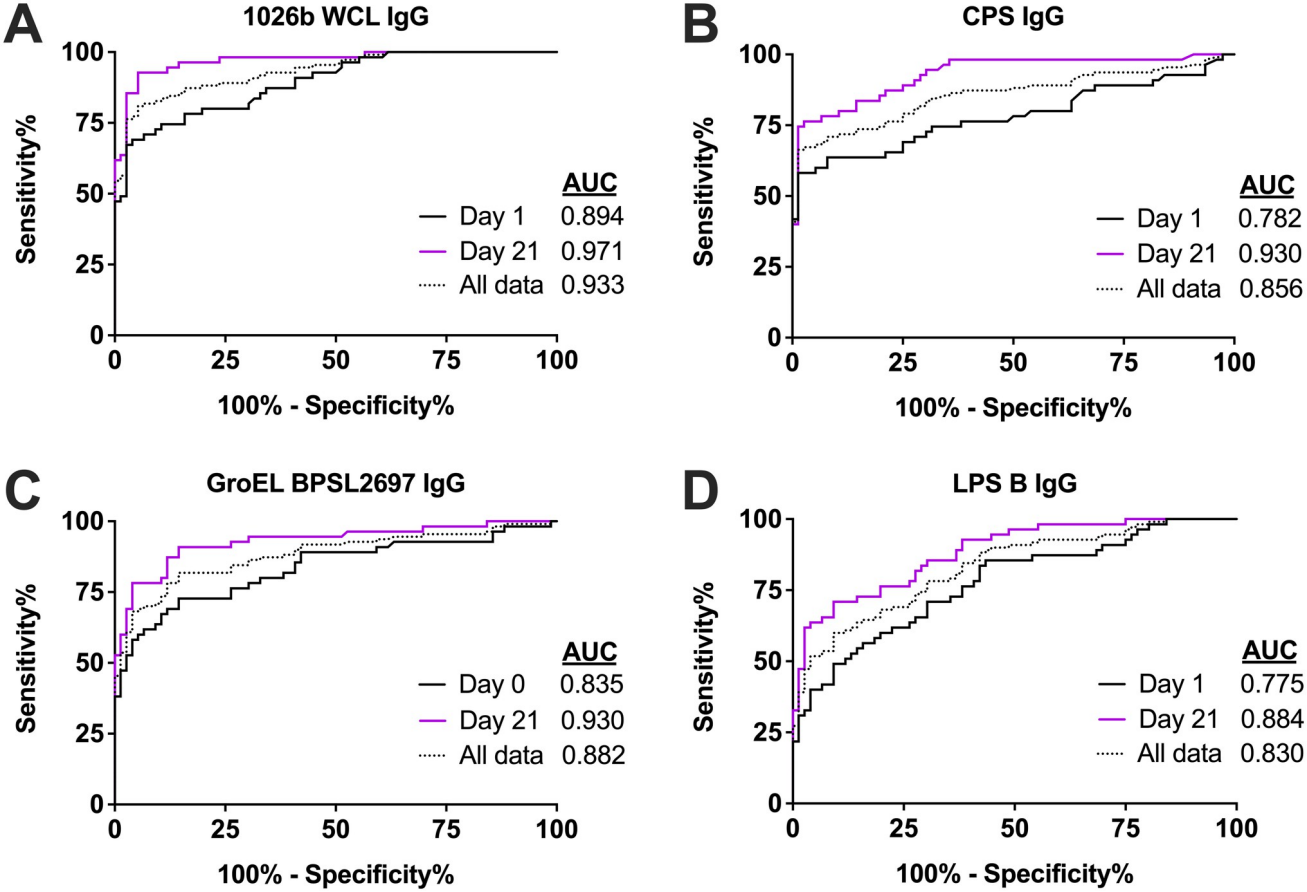
ROC analysis of *B*. *pseudomallei* lysate (multiple antigens—A) and single antigens (B-D) selected by day 21 area under the curve (AUC) analysis performance. Responses to 1026b WCL (A), CPS (B), GroEL (C), and LPS B (D) antigens are shown using non-melioidosis human sera samples compared with day 0 (black), day 21 (purple), or all melioidosis-confirmed patient samples (dashed). The area under the curve (AUC) value for each data set is shown.

We further characterized the diagnostic capacity for individual microsphere conjugated antigens using the MAGPIX reactivity data shown in Fig 2. To this end, we performed a receiver operating characteristic (ROC) curve analysis on the IgG and IgM reactive antibodies for these antigens (Fig 3). In addition, we have estimated the area under the curve (AUC) for the ROC curves (Fig 3) and summarized AUC for each antigen at the different time points (Table 2 and S2 Table). Antigens that have an AUC value close to 0.5 will not differentiate melioidosis-confirmed patient sera samples from non-melioidosis individual sera samples and a diagnostic antigen that has an AUC of 1 will perfectly differentiate these two groups. We identified 36 antigens that have an AUC of greater than 0.65 for either antibody isotype, which is at the low end of diagnostic assay potential (Table 2 and S2 Table). Within this set, the top diagnostic antibody response was IgG, which comprised eight of the top reactive antigen reactive antibody responses by day 21. In addition, the AUC for each antigen increased from day 0 (0.73 to 0.84 AUC) to day 21 (0.84 to 0.93 AUC). However, the WCL was more diagnostic compared to the top single antigens (0.971 for WCL compared to 0.93 at day 21 for CPS and BPSL2697). We next looked at the sensitivity and specificity of the individual antigens or mixtures conjugated to the microspheres (Table 3). The highest specificity ranged from ~98% for CPS and at the lowest was around ~59% for LPS depending on the assay cutoff employed. For sensitivity, the highest sensitivity was ~84% for LPS A but could be as low as 35% for the same antigen depending on the cutoff used. The sensitivity increased for each antigen when looking at sample collected later during infection. There are several individual antigens that could be used to diagnose melioidosis patients and diagnostic performance of these antigens varies.

**Table 2:** AUC Summary statistics of the top 10 individual or multiple antigens as determined by day 21 samples^1^.

Antigen	Protein Name	Detected response	AUC Negative vs					
			Day 0	Rank^2^	Day 0 95% CI	Day 21	Rank^2^	Day 21 95% CI
WCL 1026b	na	IgG	0.894	1	0.840 to 0.948	0.971	1	0.944 to 0.997
CPS	na	IgG	0.782	5	0.693 to 0.870	0.930	2	0.885 to 0.976
BPSL2697	GroEL	IgG	0.835	2	0.760 to 0.910	0.930	3	0.882 to 0.977
WCL 1026b	na	IgM	0.822	3	0.749 to 0.895	0.917	4	0.867 to 0.966
LPS B	na	IgG	0.775	6	0.694 to 0.856	0.884	5	0.828 to 0.940
CPS	na	IgM	0.739	11	0.646 to 0.831	0.881	6	0.820 to 0.941
LPS A	na	IgG	0.753	8	0.668 to 0.839	0.874	7	0.816 to 0.933
BPSS0477	GroEL2	IgG	0.794	4	0.711 to 0.876	0.863	8	0.797 to 0.930
BPSL2096	AhpC	IgG	0.727	12	0.635 to 0.820	0.852	9	0.786 to 0.918
BPSS0476	GroS	IgG	0.747	10	0.663 to 0.831	0.838	10	0.772 to 0.904

^1^ Reactivity data for the analysis is from Fig 2, which include selected melioidosis paired samples Day 0 (n = 56), Day 21 (n = 56), and non-melioidosis (n = 40).

^2^ The antigens are ranked by highest AUC at Day 21 stratified samples. The rank of day 0 stratified samples is also shown.

**Table 3:** Sensitivity and specificity of the top 10 individual antigens or WCL conjugated to Luminex microspheres^1^.

Antigen	Detected response	Cutoff (MFI)^2^	% Specificity	95% CI	% Sensitivity				All data	All data 95% CI
					Day 0	Day 0 95% CI	Day 21	Day 21 95% CI		
WCL 1026b	IgG	573	96.1	88.9 to 99.2	69.1	55.2 to 80.9	85.5	73.3 to 93.5	77.3	68.3 to 84.7
		499	94.7	87.1 to 98.6	69.1	55.2 to 80.9	92.7	82.4 to 98.0	80.9	72.3 to 87.8
CPS	IgG	572	97.4	90.8 to 99.7	58.2	44.1 to 71.4	76.4	63.0 to 86.8	67.3	57.7 to 75.9
		620	98.7	92.9 to 100.0	58.2	44.1 to 71.4	74.6	61.0 to 85.3	66.4	56.7 to 75.1
BPSL2697	IgG	979	85.5	75.6 to 92.6	72.7	59.0 to 83.9	90.9	80.1 to 97.0	81.8	73.3 to 88.5
		980	85.5	75.6 to 92.6	72.7	59.0 to 83.9	90.9	80.1 to 97.0	81.8	73.3 to 88.5
WCL 1026b	IgM	408	79.0	68.1 to 87.5	70.9	57.1 to 82.4	87.3	75.5 to 94.7	79.1	70.3 to 86.3
		466	86.8	77.1 to 93.5	58.2	44.1 to 71.4	85.5	73.3 to 93.5	72.7	63.4 to 80.8
LPS B	IgG	1070	56.6	44.7 to 67.9	85.5	73.3 to 93.5	92.7	82.4 to 98.0	56.6	44.7 to 67.9
		2605	90.8	81.9 to 96.2	49.1	35.4 to 62.9	70.9	57.1 to 82.4	60.0	50.2 to 69.2
CPS	IgM	348	92.1	83.6 to 97.1	56.4	42.3 to 69.7	72.7	59.0 to 83.9	64.6	54.9 to 73.4
		432	96.1	88.9 to 99.2	56.4	42.3 to 69.7	61.8	47.7 to 74.6	59.1	49.3 to 68.4
LPS A	IgG	969	59.2	47.3 to 70.4	74.6	61.0 to 85.3	94.6	84.9 to 98.9	84.6	76.4 to 90.7
		2408	89.5	80.3 to 95.3	27.3	16.1 to 41	43.6	30.3 to 57.7	35.5	26.6 to 45.2
BPSS0477	IgG	2490	90.8	81.9 to 96.2	62.5	48.6 to 75.1	68.6	54.1 to 80.9	65.4	55.6 to 74.4
		2077	89.5	80.3 to 95.3	62.5	48.6 to 75.1	74.5	60.4 to 85.7	68.2	58.5 to 76.9
BPSL2096	IgG	1587	98.7	92.9 to 100.0	43.6	30.3 to 57.7	50.9	37.1 to 64.7	47.3	37.7 to 57.0
		708	75.0	63.7 to 84.2	58.2	44.1 to 71.4	81.8	69.1 to 90.9	70.0	60.5 to 78.4
BPSS0476	IgG	470	61.8	50.0 to 72.8	81.8	69.1 to 90.9	94.6	84.9 to 98.9	88.2	80.6 to 93.6
		1232	89.5	80.3 to 95.3	30.9	19.1 to 44.8	45.5	32.0 to 59.5	38.2	29.1 to 47.9

^1^ Reactivity data for the analysis is from Fig 2, which include selected melioidosis paired samples Day 0 (n = 56), Day 21 (n = 56), and non-melioidosis (n = 40).

^2^ The cutoff was selected using the highest Youden’s index for the day 0 data, 21 data, or a specificity around 90%

### Modeling the data to increase assay prediction performance

**Fig 4:**
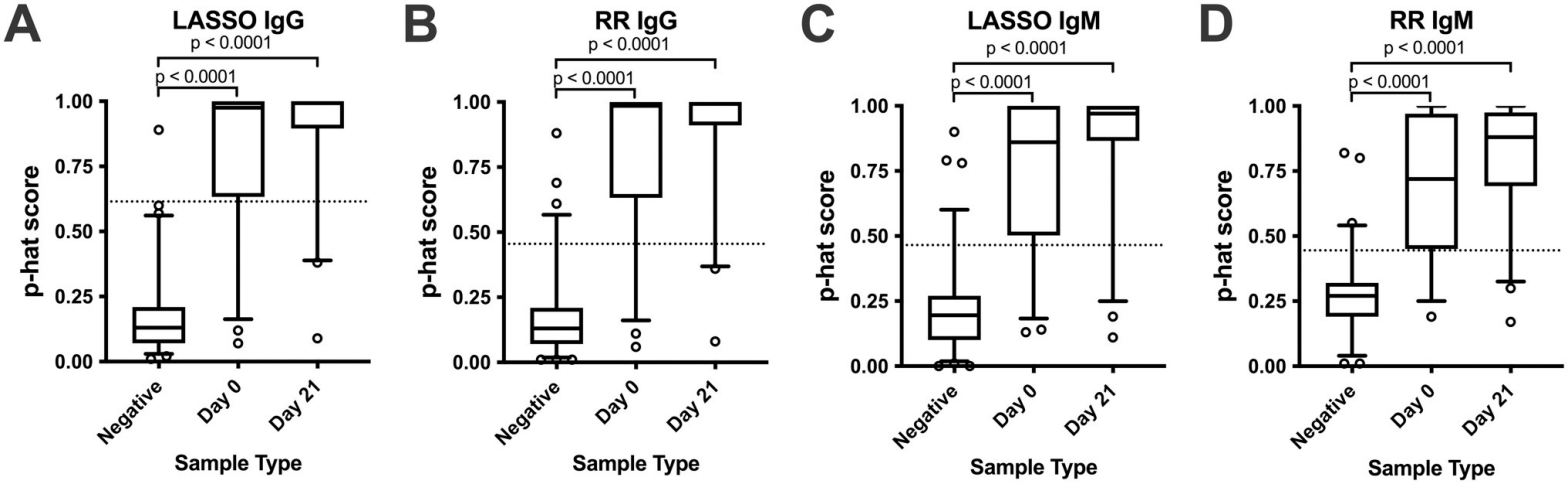
p-hat distribution using the LASSO (A and C) and Ridge Regression (RR, B and D) models. Samples were stratified based on the patient status and collection time. The models were developed using IgG (A and B) and IgM (C and D) reactivity. A $\hat{\text{p}}$ (p-hat) value of 0 is unlikely to be melioidosis and a value of 1 is likely to be melioidosis. A cutoff to call sensitivity and specificity is shown by the dotted line and corresponds to Table 4. $\hat{\text{p}}$ value significance was determined by a Kruskal-Wallis test followed by a Dunns pairwise comparison. The Day 0 vs Day 21 was not significant for all antibody isotype models.

Multiple antigens had some diagnostic potential for differentiating melioidosis patients from non-melioidosis healthy adults. However, we investigated using multiple antigens in a single model to increase the diagnostic potential of a multiplex assay. We used all data to train models (binomial logistic regression or least absolute shrinkage and selection operator (LASSO)) to determine immune responses that correlate with healthy individuals or culture confirmed melioidosis patients. Importantly, these models generate a probability score (p-hat) ranging from 0 (non-melioidosis) to 1 (melioidosis). We determined the frequency distribution of all the evaluation data using Ridge regression and the LASSO models p-hat scores (Fig 4). Regardless of the model tested or the antibody isotype tested, there was a statistical difference (P<0.0001) between the non-melioidosis sera samples and melioidosis-confirmed samples at either time point. However, there is still overlap between the melioidosis and the negative samples.

**Fig 5:**
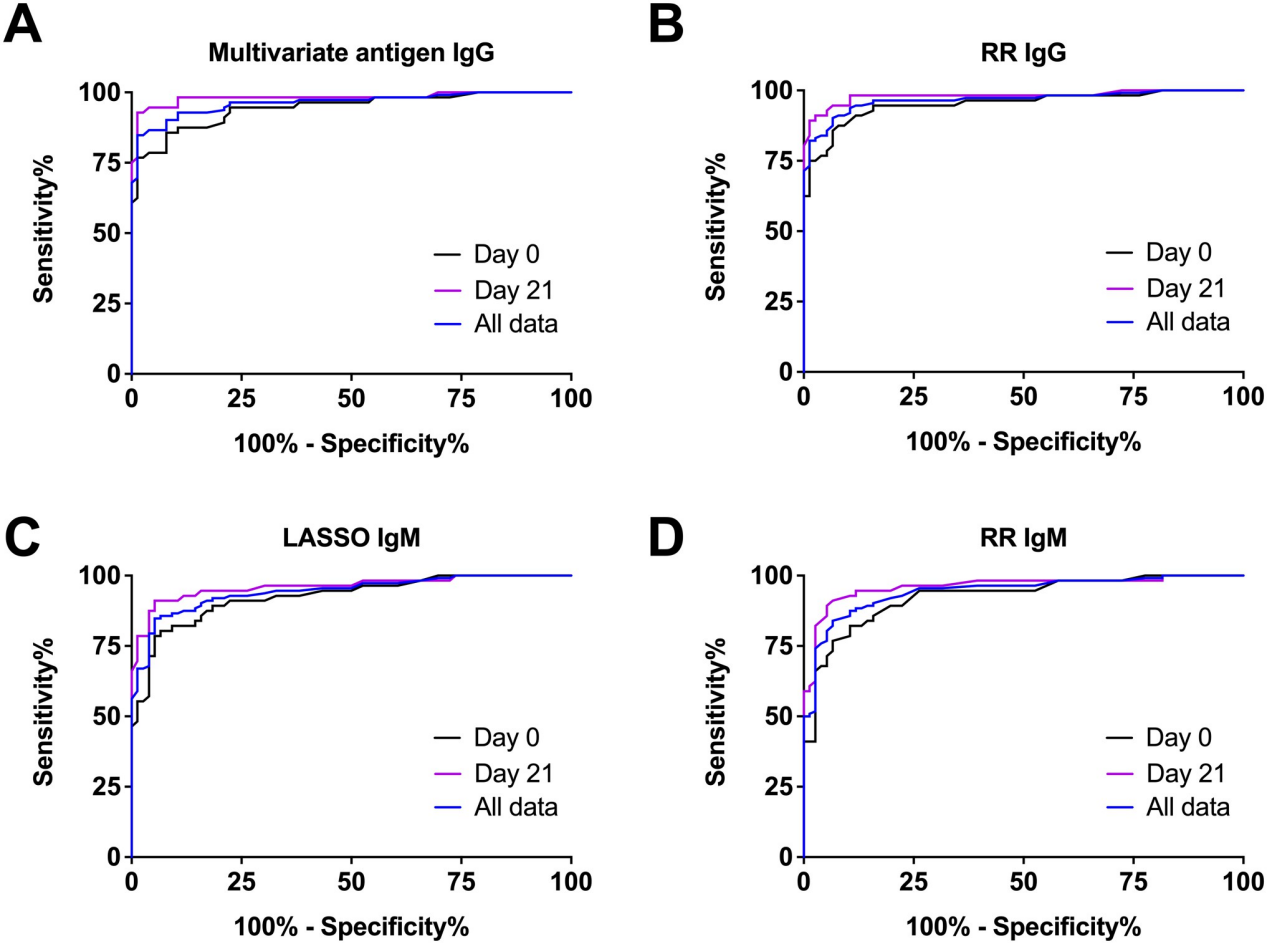
ROC analysis of the LASSO and Ridge Regression (RR) IgG and IgM models using human patient samples. A (p-hat) $\hat{\text{p}}$ score were generated from the multiple antigen reactivity (median fluorescent intensity shown in Fig 2) for IgG or IgM. n = 188 patient samples with 112 paired samples from 56 individuals.

We also evaluated all the samples by ROC curve analysis (Fig 5) and further stratified the samples into early or initial samples (day 0) or samples collected approximately 21 days later (day 21). Like the single antigens and the WCL ELISA results, the multiple antigen model did not distinguish early melioidosis samples from the negative samples as well as the day 21 samples, which suggests that the assay improves with time. We further quantified this by determining the AUC value for each curve. The values for each curve area, and the optimal sensitivity and specificity for each using the Youden’s J score are shown in Table 4. From this analysis, we observed that the worst overall model was the LASSO and RR IgM models with an AUC of 0.943. For these models, an ~84% sensitivity and 94% specificity for each IgM multivariate model was determined. When stratified, the sensitivity improved to 91% for day 21 samples. In contrast, the best model for diagnosing melioidosis samples is the RR IgG model with an AUC value of 0.966 for all samples tested. This area translated to a 90.2% sensitivity and a 93.4% specificity. When stratified, the sensitivity improved to 94% for day 21 samples. We compared this data to a single antigen within the assay tested. The best single antigen was the IgG response to CPS and this antigen had an AUC of 0.857 and a 66.6% sensitivity and a 98.7% specificity. Which suggests that a multi-antigen test can outperform a single antigen assay.

**Table 4:** Summary information for each multivariate model.

Model	samples	AUC	95% CI	Cutoff	J index^2^	% Sens	Sens 95% CI	% Spec	Spec 95% CI
LASSO IgG	All	0.963	0.938 to 0.988	0.615	0.84	84.8	76.8 to 90.9	98.7	92.9 to 100.0
	Day 0	0.945	0.905 to 0.985	0.615	ND	76.79	63.6 to 87.0		
	Day 21	0.981	0.956 to 1.006	0.615	ND	92.86	82.7 to 98.0		
RR IgG	All	0.966	0.942 to 0.990	0.455	0.84	90.2	83.1 to 95.0	93.4	85.3 to 97.8
	Day 0	0.952	0.913 to 0.990	0.455	ND	85.71	73.8 to 93.6		
	Day 21	0.981	0.955 to 1.01	0.455	ND	94.64	85.1 to 98.9		
LASSO IgM	All	0.943	0.912 to 0.974	0.465	0.8	84.8	76.8 to 90.9	94.7	87.1 to 98.6
	Day 0	0.925	0.879 to 0.971	0.465	ND	78.57	65.6 to 88.4		
	Day 21	0.961	0.927 to 0.996	0.465	ND	91.07	80.4 to 97.0		
RR IgM	All	0.943	0.911 to 0.975	0.445	0.77	83.9	75.8 to 90.2	93.4	85.3 to 97.8
	Day 0	0.925	0.878 to 0.971	0.445	ND	76.79	63.6 to 87.0		
	Day 21	0.962	0.927 to 0.996	0.445	ND	91.07	80.4 to 97.0		

The models were trained with data from Fig 2 (selected samples^1^), which include negative and Day 21 training data and they were evaluated using the same training data and the day 0 sample data.

^1^ Reactivity data for the model training is from Fig 2, which include selected melioidosis paired samples Day 0 (n = 56), Day 21 (n = 56), and non-melioidosis (n = 40).

^2^ ND = not determined

Finally, we determined the antigens that contributed to each IgG or IgM isotype model, which will help identify if a subset of the 46 antigens were required for increased assay performance. Table 5 shows antigens that statistically contributed to the evaluation models using the 46 evaluated antigens. For the standard binomial regression, antigens that had a P<0.05 are shown. The s0 listed in Table 5 is the standardized coefficient for each antigen as evaluated by the LASSO method and a larger magnitude is more influential to the model. Interestingly, these models did not use the same number of antigens or even the same antigens in differentiating melioidosis patients from healthy donors. For IgM, there were three antigens that contributed significantly to the RR model and eight antigens that contributed to the LASSO model. For IgG, 16 antigens were used for the RR model and eight antigens for the LASSO model. In cumulation, all 46 antigens are not required for an improved diagnostic assay performance and a smaller subset may be sufficient.

**Table 5:** Antigens and the associated antibody response that contributed to a binomial or LASSO logistic regression models^1^.

number	Ab Response	Antigen locus tag, name, or antigen mixture	Common name^2^	Binomial adj p-value	LASSO s0
1	IgM	MSHR5855 WCL	NA	7.36E-18	0.00011
2	IgM	BPSL2827	DNAk	>0.05	8.22E-05
3	IgG	BPSL1404	ClpX	>0.05	6.82E-05
4	IgG	BPSL1201	IPMS	7.78E-07	5.42E-05
5	IgG	MSHR5855 WCL	NA	7.59E-25	5.31E-05
6	IgM	BPSL0739	Hypothetical	>0.05	3.67E-05
7	IgG	LPSB	LPSB	1.50E-14	3.58E-05
8	IgG	BPSL1743	Arg	2.38E-08	2.83E-05
9	IgM	BPSL3222	rpIL	>0.05	2.70E-05
10	IgG	CPS	CPS	1.98E-18	2.34E-05
11	IgG	BPSL2697	GroEL	5.46E-20	1.65E-05
12	IgM	BPSS1850	Hypothetical	>0.05	1.44E-05
13	IgM	BPSL2096	AhpC	7.43E-05	1.17E-05
14	IgM	BPSL3396	AtpD	>0.05	8.96E-06
15	IgG	BPSS0476	GroS	1.35E-05	2.35E-06
16	IgM	BPSL2522	OmpA	>0.05	1.68E-06
17	IgM	CPS	CPS	3.89E-14	0
18	IgG	BPSL2096	AhpC	5.06E-13	0
19	IgG	BPSS0477	GroEL2	3.10E-12	0
20	IgG	LPSA	LPSA	7.58E-11	0
21	IgM	BPSL2697	GroEL	2.84E-08	0
22	IgG	BPSS1652	ABC trans	1.40E-06	0
23	IgG	BPSS0530	Hypothetical	2.00E-06	0
24	IgG	BPSL0739	Hypothetical	3.97E-06	0
25	IgG	BPSS0135	Hypothetical	8.46E-06	0
26	IgG	BPSL2522	OmpA	1.03E-05	0
27	IgG	BPSS1769	NADH	9.19E-05	0

The IgG and IgM response were merged into a single model for the multivariate statistic evaluation.

^1^ IgG or IgM LASSO models were developed using the data generated in Table 4.

^2^ NA = Not available, Hypothetical = Hypothetical protein

### Analysis of the multivariate models using the evaluation data and comparison to single antigens and the IHA assay

**Fig 6:**
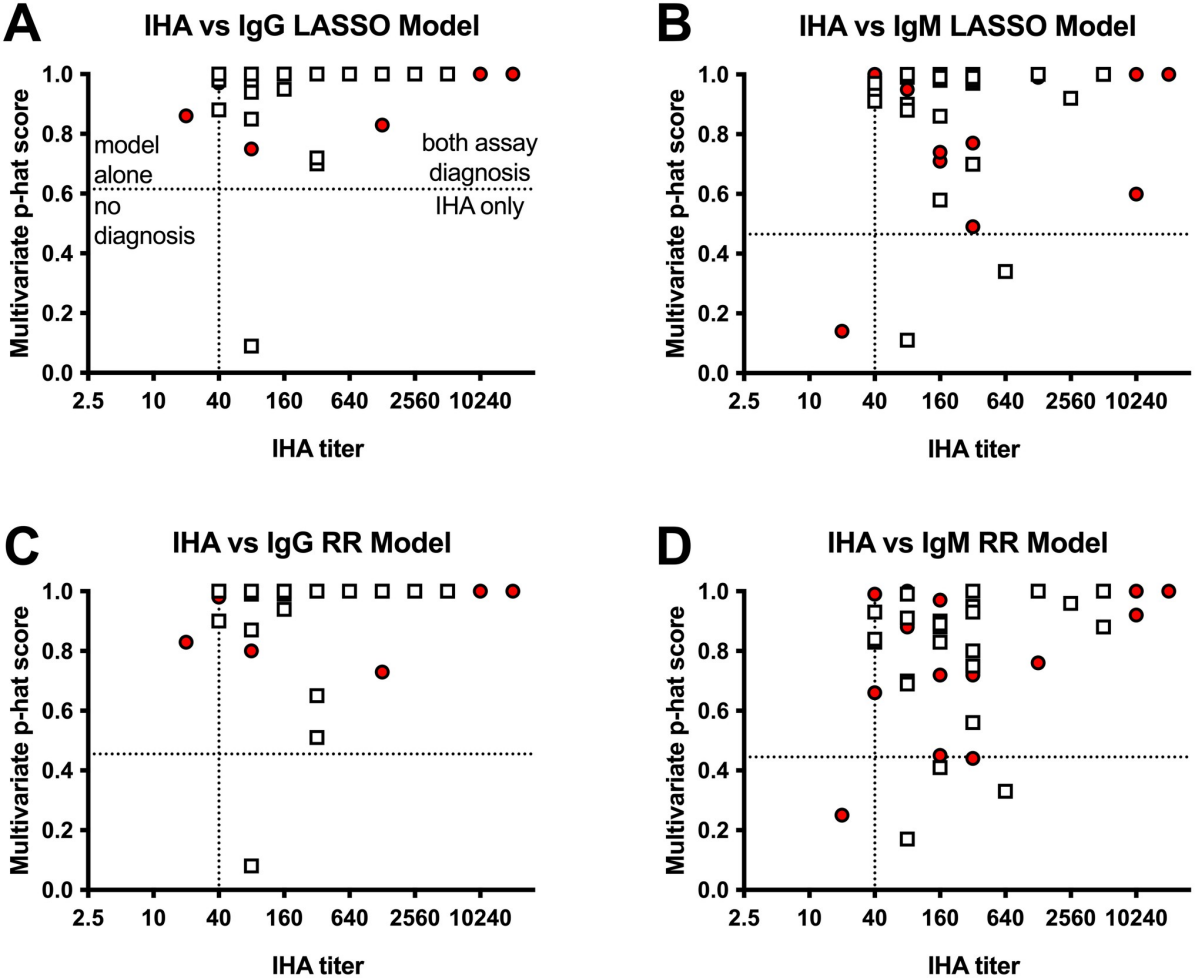
Comparison of the IHA results to the multivariate serology assay. IHA results (X-axis) and the multivariate p-hat probability score (Y-axis) are shown for the same sample collected from culture confirmed melioidosis patients. The different multivariate models are shown in separate panels (A-D). The day 0 (red circle) and day 21 (white squares) are shown. The cutoff for the IHA assay is 1:40 and the multivariate model cut-offs are consistent as shown in Table 5. The interpretation of the cutoff panels and which assay would correctly diagnose melioidosis is shown in panel A. A total of 40 samples that have both IHA and multivariate model values were compared.

We compared the multivariate assay with the historically accepted IHA assay. IHA data was collected from a subset of the human samples during patient treatment and were also analyzed by the MAGPIX. We performed a regression analysis (Fig 6) to illustrate how the assays were performing. Using this comparison, the multivariate IgG assays diagnosed 95% of the similar diagnosed samples using an IHA cut off ≥1:40 titer. However, each assay diagnosed one sample that the other assay did not diagnose (2.5%). The multiple antigen IgM model did not perform as well when detecting melioidosis patients compared to the IHA assay. IgM is the primary antibody detected by the IHA [42] and we thought the IHA would be consistent with the IgM multivariate assays. However, the multiple antigen assay failed to call 5–10% of the samples that the IHA properly called, which suggests that we are not using or weighting antigens properly in the model that the IHA primarily utilizes for melioidosis patient diagnosis. In combination, the data suggest that the IgG multivariate assays have good diagnostic potential.

## Discussion

Efforts continue to develop serological assays for melioidosis that improve on the sensitivity and specificity of the IHA. Several serological assays have been developed to detect melioidosis using single antigens or complex mixtures [12–22]. This study shows that a multiplex serology assay using an antigen weighted model increases assay performance to interpret infection status compared to single antigens. Specifically, CPS, the best single antigen, had an ROC AUC of 0.856 (Fig 3) and this increased to 0.966 (Table 4) for the best multiple antigen model. In addition, the multiple antigen model performed comparably to the IHA using a limited data set. Thus, multiple antigen data detection can potentially increase diagnostic assay performance for melioidosis diagnosis.

To allow for prompt treatment, important factors for a diagnostic assay are sensitivity with high specificity at early times after admission to hospital settings. In addition, a highly specific assay needs to also be considered in non-endemic regions with sporadic *B*. *pseudomallei* infections due to travel or reduced environmental sources. Single antigens and the WCL microspheres on the MAGPIX platform had some melioidosis diagnostic potential at both time points. The WCL microsphere generated a specificity of 95% and had a sensitivity of approximately 69% for the initial sample (Day 0). However, the multiple antigen reactivity and model increased sensitivity to >85% when using the initial sample and a >94% sensitivity ~21 days later. Importantly, the overall specificity was >93%. It is important to note that the multiple antigen model was developed using the same antigens that had lower sensitivity and specificity and by using the antigens in combination within a model can improve the performance of an assay. These data suggest that a multiplex assay can increase sensitivity of assays to detect melioidosis, while keeping specificity high.

Single antigen ELISAs have greatly improved diagnosing melioidosis in a research setting or CLIA based setting, decreasing the overall cost of assay development. In many cases the antigen plays a very important role in differentiating melioidosis from negative samples. To date, the best single performing antigens are *B*. *pseudomallei* Hcp1 and LPS A. Depending on the antigen, these assays were reportedly 72–83% sensitive and 94–100% specific using melioidosis patient samples from Thailand and negative samples from the United States [13,19]. However, the sensitivity decreased to 48–63% for single antigens or antigen assay combinations when restricting sample collection to 24 hours after patient admission [17]. In this report, we screened 46 antigens and a whole cell lysate pool for single antigen using a the MAGPIX multiplex platform. These antigens did not include Hcp1, but the assay performed slightly better than the Hcp1 assay overall and for early sample collection times. However, these results should be interpreted with caution due to different samples used between the two studies. It will be important in future multiplex studies to include Hcp1 and to include serum samples from additional endemic regions to determine the optimum assay performance.

As previously mentioned, LPS has been used in ELISA assays with melioidosis diagnostic performance [13]. Surprisingly, in this study the LPS A had a low specificity or sensitivity depending on the assay cutoff, being much lower than what was previously reported for this antigen in ELISA assays [13]. In addition, LPS A was not used in the LASSO model but was used in the RR model (Table 5). It has been reported that LPS is a major target of the antibody response in WCL ELISA [19]. We prescreened the non-melioidosis, non-endemic control samples for cross reactivity to the WCL ELISA and used a higher proportion of the reactive samples in the assay evaluation than represented by natural frequencies (Fig 1). These reactive samples had a higher LPS A and B signal compared to the non-reactive negative samples (Fig 2). These samples were collected in the United States where melioidosis is rare and usually reflects imported disease from infection with *B*. *pseudomallei* acquired overseas in endemic countries. The higher reactivity suggests that other pathogen carbohydrates are generating antibody responses that can cross react with *B*. *pseudomallei* and could contribute to false-positive responses.

Given the difficulties with culture and identification of *B*. *pseudomallei* in many endemic and non-endemic regions for melioidosis, alternative laboratory techniques can facilitate a diagnosis of “probable melioidosis” and support initiation of the potentially lifesaving specific therapy required [2]. One promising technology is the use of rapid antigen testing with a lateral flow immunoassay strip for direct analysis of patient clinical samples such as blood, urine, sputum, and pus for the presence of *B*. *pseudomallei* capsular polysaccharide [43]. Nevertheless, sensitivity of this test on blood is low, and there remains a role for serology assays. One possibility to enhance diagnosis where bacterial culture is problematic or where culture has been initially negative but melioidosis remains a diagnostic possibility, is combining serology with antigen detection, as reported in a recent study [17]. For such circumstances, the optimization of the serology assay used is critical.

The further development of a multiplex serology assay using an antigen weighted multiple antigen model for antibody reactivity from multiple *B*. *pseudomallei* antigens has the potential to optimize both sensitivity and specificity for the serological diagnosis of melioidosis. We envision that we could decrease the number of reactive antigens that are included in a final test that can still elevate sensitivity and specificity compared to single antigens. Further modification of the assay development time could also decrease the overall time to assay results and allow prompt treatment in a clinical setting. Once validated, a smaller set of antigen conjugated microspheres would be produced and stabilized for longer term storage. Prior to performing the assay, beads would be prepared prior to use and developed using clinically collected sera samples. Many of the final steps for assay distribution would require the commercialization and FDA approval of the assay. This will allow the assay to be performed outside of research only use or CLIA setting and provide the improvements in melioidosis diagnosis that are needed.

## Supporting information

S1 Fig Frequency distribution of samples used for MAGPIX evaluation. The distribution of the samples is shown by counting the number of serum samples used within a bin of every 2 days starting at day zero. Collection of the initial sample (blue line) or relative to days after admission to the hospital (red line) are shown. Samples were selected based on the proximity to the collection of the initial sample (day 0). We selected the next closest serial sample that was approximately 21 days later. A total of 56 melioidosis-confirmed patients are shown with a total of 112 serum samples.
(TIF)

S1 Table Purified antigen quantity and purity. (XLSX)

S2 Table Summary statistics of the individual antigens or antigen mixtures. na = not applicable. Antigens for each antibody isotype are sorted from the highest to lowest AUC on day 21.
(XLSX)

S3 Table MAGPIX Median Florescence data. DPMS = Darwin Prospective Melioidosis Study.
(XLSX)
