# Supplementary figures and images for: Development and evaluation of a multiplex serodiagnostic bead assay (BurkPx) for accurate melioidosis diagnosis
Source: PLoS Negl Trop Dis. 2023 Feb 8;17(2):e0011072. doi: 10.1371/journal.pntd.0011072 (PMC9907819; doi:10.1371/journal.pntd.0011072)

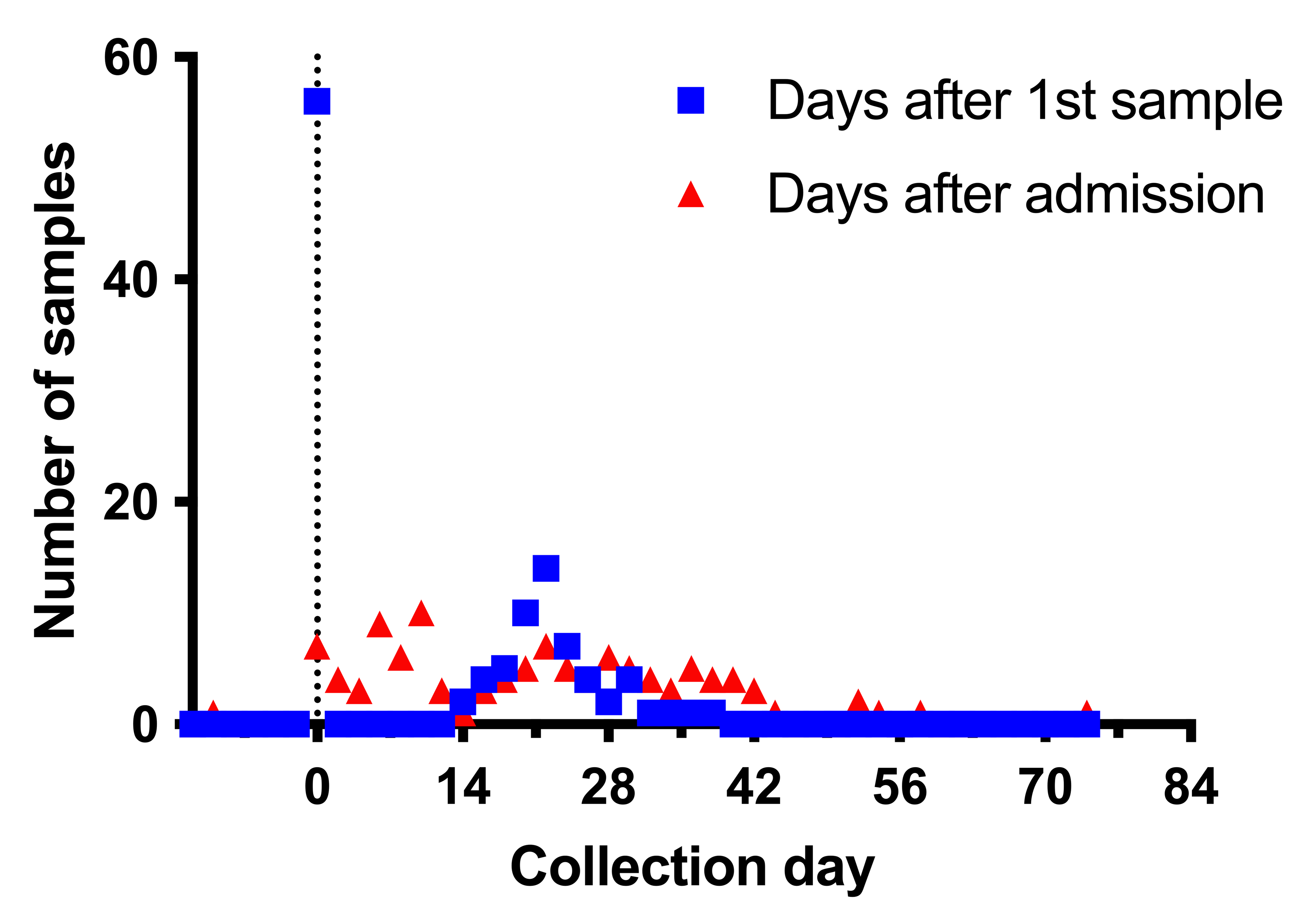

Supplement: S1 Fig — The distribution of the samples is shown by counting the number of serum samples used within a bin of every 2 days starting at day zero. Collection of the initial sample (blue line) or relative to days after admission to the hospital (red line) are shown. Samples were selected based on the proximity to the collection of the initial sample (day 0). We selected the next closest serial sample that was approximately 21 days later. A total of 56 melioidosis-confirmed patients are shown with a total of 112 serum samples. (TIF) [file pntd.0011072.s001.tif]
